# Supplementary material for: A lab-based test of the gravitational redshift with a miniature clock network
Source: Nat Commun. 2023 Aug 12;14:4886. doi: 10.1038/s41467-023-40629-8 (PMC10423269; doi:10.1038/s41467-023-40629-8)
Supplement: Supplementary file 1 — Supplementary Information [file 41467_2023_40629_MOESM1_ESM.pdf]

Supplementary Information for "A lab-based test of the gravitational redshift with a miniature clock network"

Xin Zheng[1,2,†], Jonathan Dolde[1,†], Matthew C. Cambria[1], Hong Ming Lim[1], Shimon Kolkowitz[1,2,*]

[1] *Department of Physics, University of Wisconsin-Madison, WI 53706, USA*

[2] *Department of Physics, University of California, Berkeley, CA 94720, USA*

[†] *These authors contributed equally to this work*

[*] *To whom correspondence should be addressed; E-mail: kolkowitz@berkeley.edu*

SUPPLEMENTARY NOTE 1: ATOMIC TEMPERATURES

Representative data from clock sideband thermometry on the $|g\rangle \leftrightarrow |e\rangle$ transition at 130 E_{rec} is shown in Supplementary Figure 1a. The reduced height of the red sideband ($n_z \to n_z - 1$, where n_z is the axial vibrational quantum number) indicates that $> 99\%$ of the atoms are populated in the lattice ground band ($n_z = 0$). A separate clock probe perpendicular to the lattice axis taken at 15 E_{rec} is shown in Supplementary Figure 1b, in which the Doppler broadened profile indicates a radial atomic temperature below 200 nK.

SUPPLEMENTARY NOTE 2: ATOMIC COHERENCE TIMES

We use a synchronized Ramsey sequence to measure the atomic coherence. We prepare two atomic ensembles in the $|e, m_F = +3/2\rangle$ state and apply a $\pi/2$ pulse on the $|e, m_F = +3/2\rangle \leftrightarrow |g, m_F = +5/2\rangle$ transition. After waiting for a varying dark time ranging from 100 ms to 40 s, we apply a second $\pi/2$ pulse with a random phase relative to the first. We then measure the excitation fractions of each ensemble and plot the averaged contrast as a function of dark times. We observe an exponential decay of the contrast with a time constant of 32.1(9) s at 15 E_{rec} (Supplementary Figure 2), 300 times longer than the measured atom-laser coherence time ($\sim$ 100 ms). We note that while the atomic coherence time is about 30 s, the typical clock interrogation time in our measurements is around 10 s, chosen to minimize the QPN because lost of atoms and reduced contrast at longer dark times [1].

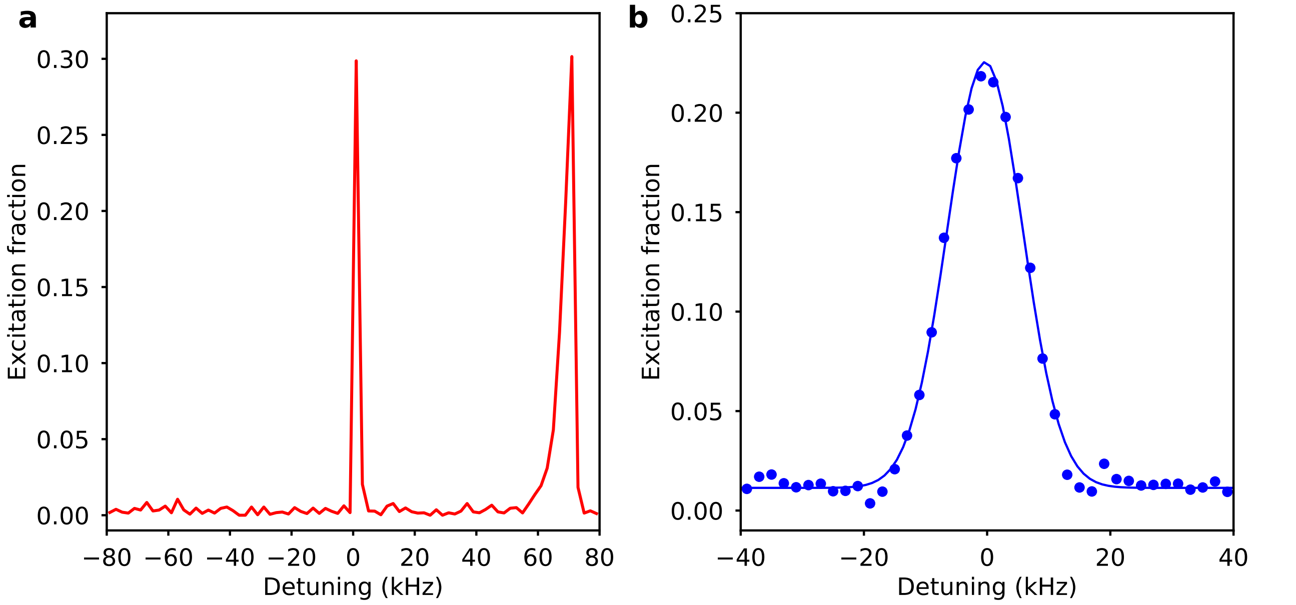

Supplementary Figure 1. Atomic temperatures. (a) Axial clock sideband thermometry taken at 130 $E_{\rm rec}$ lattice trap depth following in-lattice cooling. The reduced height of the red sideband ($n_z \rightarrow n_z - 1$) indicates that 99% of atoms are populated in the lattice ground band ($n_z = 0$). (b) Radial Doppler broadened profile taken at 15 $E_{\rm rec}$. A Gaussian fit (solid blue line) yields a radial temperature of 180 nK.

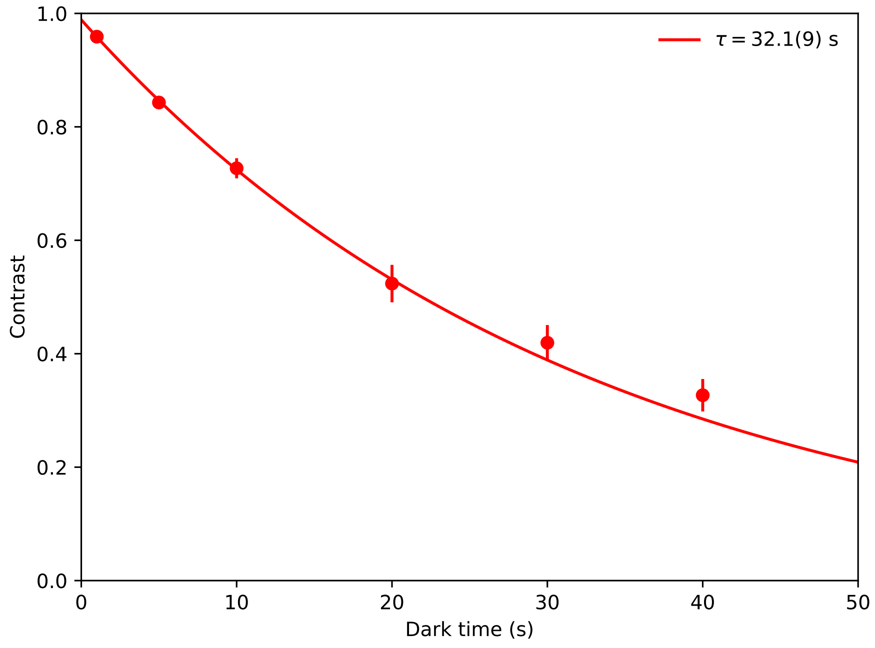

Supplementary Figure 2. Atomic coherence times. Decay of Ramsey contrast as a function of dark times at $u_{op} = 15\ E_{\rm rec}$ (scatter points). An exponential fit (solid line) yields an atomic coherence time of 32.1(9) s.

SUPPLEMENTARY NOTE 3: SYSTEMATIC EVALUATION

A. Zeeman shifts

Coupling of the clock states to external magnetic fields give rise to Zeeman shifts of the clock transition frequency. For a single atomic ensemble with magnetic field amplitude B, the Zeeman shifts when probing the $|g, m_F = \pm 5/2\rangle \leftrightarrow |e, m_F = \pm 3/2\rangle$ clock transitions can be expressed as

$$\nu_{\mathrm{ZS}}(B, \pm) = \pm\mu_L B + \mu_Q B^2, \tag{1}$$

where μ_L and μ_Q are the first and second-order Zeeman shift coefficients, respectively. For differential clock comparison between ensemble pair (i, j), where $j > i$, with differing magnetic field strengths B_i and B_j, the differential Zeeman shifts, $\Delta\nu_{\mathrm{ZS}}(B_i, B_j, \pm) = \nu_{\mathrm{ZS}}(B_j, \pm) - \nu_{\mathrm{ZS}}(B_i, \pm)$, are given by

$$\begin{aligned} \Delta\nu_{\mathrm{ZS}}(B_i, B_j, \pm) &= \pm\mu_L(B_j - B_i) + \mu_Q(B_j^2 - B_i^2), \\ &= \pm\mu_L \delta B + 2\mu_Q B \delta B, \end{aligned} \tag{2}$$

where $B = (B_i + B_j)/2$ is the mean magnetic field amplitude, and $\delta B = B_j - B_i$ is the magnetic field difference. The first-order differential Zeeman shift (on the order of $\pm 8 \times 10^{-17}$/cm) is rejected by averaging opposite spin state transitions, while their splitting yields δB. In the limit of $|\delta B| \ll B$, the second-order differential Zeeman shift can be approximated as

$$\begin{aligned} \delta B &= \Delta\nu_{\mathrm{split}}/(2\mu_L), \\ \Delta\nu_{\mathrm{ZS,2}} &= \xi \Delta\nu_{\mathrm{split}} B, \end{aligned} \tag{3}$$

where $\xi = \mu_Q/\mu_L$, and $\Delta\nu_{\mathrm{split}}$ is the splitting between the transitions with opposite spin states after subtraction of residual vector AC Stark shift.

The second-order Zeeman shift correction is applied for each measurement run, with uncertainty primarily limited by ξ, which is found to be 0.0105(1) G^{-1} by varying B and δB. This is in good agreement with the theoretical value of 0.0104(2), calculated using $\mu_L = -22.4$ Hz/G and $\mu_Q = -0.233(5)$ Hz/G^2 [2]. The bias magnetic field B is calibrated each measurement day using the magnetically sensitive transitions $|^1S_0, m_F = \pm 9/2\rangle \rightarrow |^3P_1, m_F = \pm 11/2\rangle$, and has a typical value of ~ 5.5 G with fractional uncertainty below 10^{-4}. The field difference δB (~ 1.5 mG/cm) is extracted from the splitting between the clock transitions with opposite nuclear spin states with fractional uncertainty below 10^{-3} after subtraction of the vector AC Stark shift. Through weighted averaging of the data taken at normal operating conditions, we find the mean second-order Zeeman gradient to be $-95.3(1.0) \times 10^{-19}$/cm (Fig. 2b in the main text).

B. Density shift

Due to the Pauli-exclusion principle, s-wave interactions are forbidden for identical Fermionic atoms within a single lattice site, while p-wave collisions are allowed, leading to a clock frequency shift that scales linearly with atomic density [3, 4]. In our system, the differential density shift is evaluated by varying the atom numbers loaded into each ensemble for each individual pairwise comparison. For a symmetric pair (2, 4), we find a linear slope of $-0.7(1) \times 10^{-19}$ per 100 atom number difference at our operational lattice trap depth $u_{op} = 15\ E_{\mathrm{rec}}$ (Fig. 2a in the main text). In addition, we found a weak trap volume dependence due to the Gaussian nature of the lattice

beam (less than 15 % across the entire array), which is accounted for in our evaluation as a different dependence on atom number difference for each pairwise comparison. For a typical run of the experiment, each atomic ensemble has about 2000 atoms, corresponding to an overall density shift of about $-14(2) \times 10^{-19}$. The differential density shift is suppressed by a factor of 10 when the atom number difference is bounded below 200 through optimization of ensemble loading times and conditions. In addition, we measure the atom number in each ensemble in every shot of the experiment, and the average differential density shift is calculated and corrected individually for each pairwise clock comparison measurement run. Uncertainty of density shift correction arises from shot-to-shot atom number fluctuations, for which we estimate an upper bound limit of 1×10^{-19}.

A recent study found that operation in a gravity-tilted shallow lattice allows for cancellation of density shifts at a "magic" lattice depth near 12 E_{rec}, where the partially delocalized Wannier-Stark states enable tunability of on-site p-wave versus neighbouring-site s-wave atomic interactions [5]. In this work we did not observe such a cancellation effect at shallower lattice depths, likely due to the difference in dynamics between Rabi spectroscopy as was employed in Ref. [5] versus our use of Ramsey spectroscopy with a 50:50 superposition [6]. This offers the prospect of further reducing uncertainty from differential density shifts in future works.

C. Black body radiation shift

In our experiment, the optical lattice is orientated nearly vertically, with a tilt of about 5°, and is centered with respect to the science chamber to the best of abilities. The tilt is determined by measuring the Wannier-Stark ladder resonances (see the "Wannier-Stark ladder and the expected gravitational redshift" section below for details). The recessed high-emissivity Fused Silica viewports (MPF Products) mounted on the top and bottom sides of the science chamber are the closest surfaces to the atoms, and are primarily responsible for the black body radiation (BBR) gradient along the lattice axis. To study the BBR effect [7, 8], we heat up either the top or bottom stainless steel flange of the science chamber, which results in a temperature difference of up to ± 1 K between the top and bottom viewports (see inset of Fig. 2c in the main text.

The BBR shift due to thermal gradients between the top and bottom viewports can be expressed as [9]:

$$\nu_{\text{BBR}}(z) = -2\frac{\alpha_{\text{Sr}}\sigma_{\text{SB}}}{h\epsilon_0 c} \sum_{k=\text{top,bot}} \frac{\Omega_k(z)}{4\pi} T_k^4, \tag{4}$$

where h is the Planck constant, α_{Sr} is the atom's DC polarizability, σ_{SB} is the Stefan-Boltzmann constant, $\Omega_k(z)$ corresponds to the solid angle as seen by the atom ensemble at z, and T_k denotes the temperature on the surface of the viewport. The differential BBR shift between an ensemble pair at (z_i, z_j) is then given by

$$\Delta\nu_{\text{BBR}}(z_j, z_i) = -2\frac{\alpha_{\text{Sr}}\sigma_{\text{SB}}}{h\epsilon_0 c} \sum_{k=\text{top,bot}} \left(\frac{\Omega_k(z_j)}{4\pi} - \frac{\Omega_k(z_i)}{4\pi}\right) T_k^4. \tag{5}$$

Because the viewport separation ($2Z_0 = 15$ cm) is much larger than the separation between the ensemble pairs ($\delta z_{ji} = z_j - z_i \leq 1$ cm), we approximate the solid angle difference as

$$\begin{aligned} \Delta\Omega_{ji,\text{top}} &= \pi r^2 \left(\frac{1}{(Z_0 + z_j)^2} - \frac{1}{(Z_0 + z_i)^2}\right) \approx -\frac{\pi r^2}{Z_0^3}\delta z_{ji}, \\ \Delta\Omega_{ji,\text{bot}} &= \pi r^2 \left(\frac{1}{(Z_0 - z_j)^2} - \frac{1}{(Z_0 - z_i)^2}\right) \approx \frac{\pi r^2}{Z_0^3}\delta z_{ji}, \end{aligned} \tag{6}$$

where $r = 5.7$ cm is the radius of the viewport. The differential BBR shift can be further simplified through a Taylor expansion up to order $(\delta T/T)^3$, where $\delta T = T_{\rm top} - T_{\rm bot}$ is the temperature difference between the top and bottom viewports:

$$\begin{aligned}\Delta\nu_{\mathrm{BBR},ji} &= -2\frac{\alpha_{\rm Sr}\sigma_{\rm SB}}{h\epsilon_0 c}\frac{r^2}{4Z_0^3}\delta z_{ji}T^4\left[\left(1+\frac{\delta T}{T}\right)^4 - 1\right] \\ &\approx -2\frac{\alpha_{\rm Sr}\sigma_{\rm SB}}{h\epsilon_0 c}\frac{r^2}{4Z_0^3}\delta z_{ji}T^4\left[4\left(\frac{\delta T}{T}\right)+6\left(\frac{\delta T}{T}\right)^2+4\left(\frac{\delta T}{T}\right)^3+\mathcal{O}\left(\left(\frac{\delta T}{T}\right)^4\right)\right].\end{aligned} \tag{7}$$

Because the absolute temperature ($T = 300$ K) is a factor of 150× greater than the temperature difference we applied to the viewports ($\delta T \leq 2$ K), the magnitude of the first-order term is roughly a factor of 100× larger than the second-order term. Therefore, we can approximate Eq. 7 as an expression with a linear scaling with both δT and δz_{ji}:

$$\Delta\nu_{\mathrm{BBR},ji} \approx -2\frac{\alpha_{\rm Sr}\sigma_{\rm SB}}{h\epsilon_0 c}\frac{r^2}{Z_0^3}T^3 \times \delta z_{ji} \times \delta T. \tag{8}$$

Measuring the frequency differences of the 10 ensemble pairs simultaneously, we find that as expected from Eq. 8, the resulting frequency shifts scale linearly with the temperature difference, and the corresponding slopes scale roughly linearly with the height differences (Supplementary Figure 3). Through a linear fit to the extracted slopes as a function of height differences, we find the BBR sensitivity in our system to be $-4.2(1) \times 10^{-18}$/cm per 1 K difference (Fig. 2c in the main text).

To monitor the temperature, we use commercially available 10 kΩ negative temperature coefficient thermistors (Amphenol Thermometrics, MC65F103A), rated for an interchangeability of 50 mK by the manufacturer. Relative accuracy of the temperature sensors is calibrated in an ice water bath, and we find the temperature differences between each sensor consistent within 25 mK. The temperature sensors are mounted on the stainless steel Conflat flanges of the top and bottom viewports, as well as the side flanges of the science chamber for monitoring the radial temperature inhomogeneity. Under normal operating conditions, we find the temperature difference between the top and bottom sensors lies within a range of 350 ± 100 mK and find the radial temperature inhomogeneity to be less than 75 mK.

The MOT coils are mounted in the recessed window of the science chamber, which could potentially introduce temporal thermal drifts during the sample preparation stage. To suppress this, we opt for compact coils with efficient water cooling through hollow wires, keep the ${}^1S_0 \leftrightarrow {}^1P_1$ first-stage MOT loading time (< 0.5 s) short compared to the experimental cycle time (12 s), and ensure that the duty-cycles remain consistent throughout the measurements.

While the strontium oven atom source (AOSense) is another potential source of BBR shift, the atom ensembles have no direct line of sight to the BBR photons from the oven thanks to the use of a 2D-MOT for atomic beam deflection. We operate the oven at the lowest possible temperature ($T = 360$ °C) under normal conditions, and find no statistically significant change in the frequency differences across the ensemble array at the 1×10^{-19}/cm level when intentionally increasing the oven temperature to 460 °C, a lever arm of 1.8 due to T^4 scaling bounds the systematic gradient from the oven to below 5.5×10^{-20}/cm.

Overall, for measurements taken under normal operations, we find the averaged temperature difference between top and bottom sensors to be 370(35) mK, resulting in a BBR gradient of $-15.7(1.5) \times 10^{-19}$/cm.

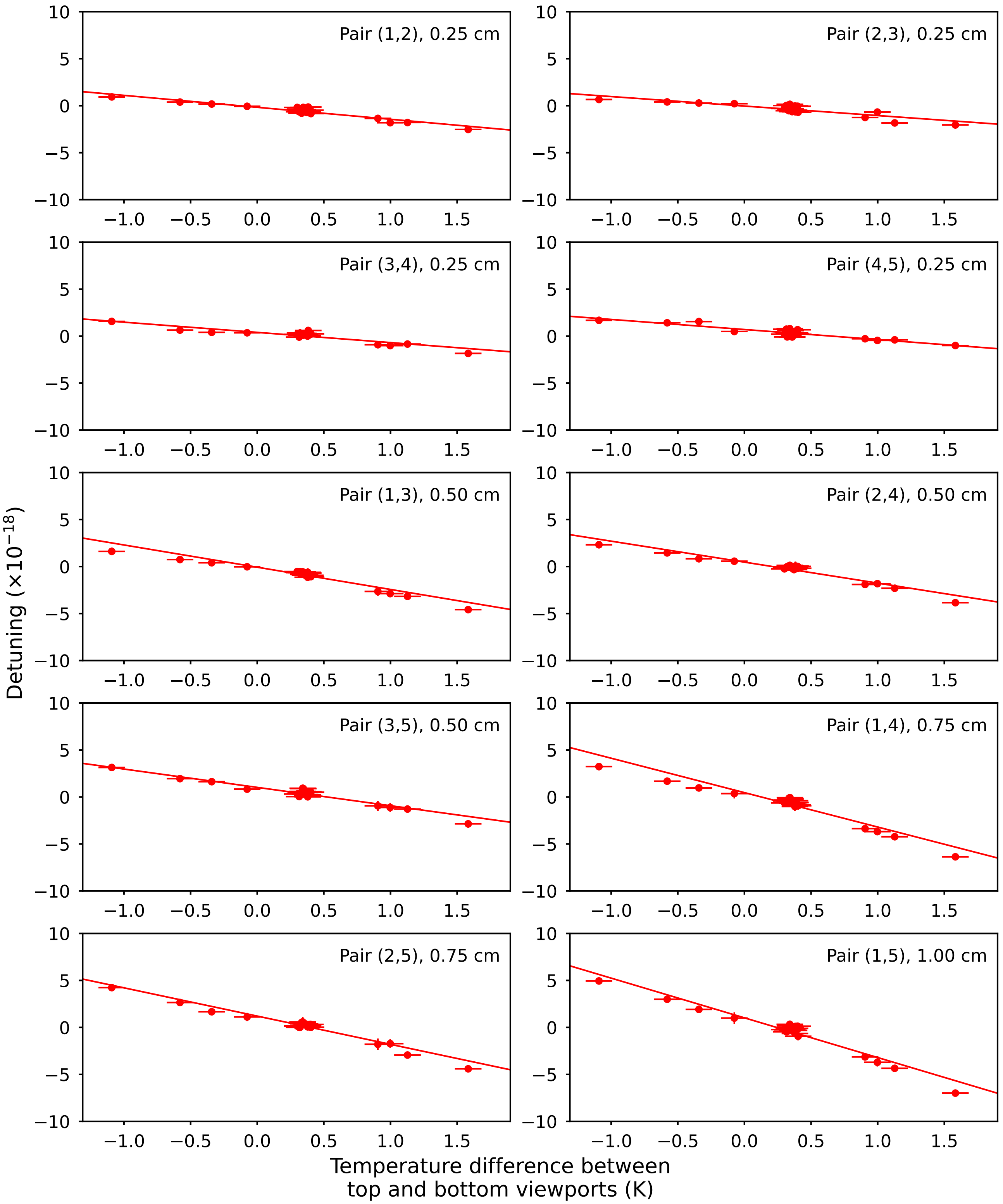

Supplementary Figure 3. Black body radiation shift evaluation. Evaluations of BBR shift for all 10 ensemble pairs simultaneously. Solid lines are linear fits to the scatter points. The y-axis scales are kept the same for all the plots. The dense data points around 0.35 K correspond to temperature differences recorded under normal operating conditions. The inset text list the ensemble pair indices and corresponding height difference.

D. Lattice light shift

The lattice light shift for a single atomic ensemble is given by [10]:

$$
\begin{aligned}
\nu_{LS}(u,\delta_L,n_z) \approx & \left(\frac{\partial\tilde{\alpha}^{E1}}{\partial\nu}\delta_L - \tilde{\alpha}^{qm}\right)\left(n_z+\frac{1}{2}\right)u^{1/2} \\
& - \left[\frac{\partial\tilde{\alpha}^{E1}}{\partial\nu}\delta_L + \frac{3}{2}\tilde{\beta}\left(n_z^2+n_z+\frac{1}{2}\right)\right]u \\
& + 2\tilde{\beta}\left(n_z+\frac{1}{2}\right)u^{3/2} - \tilde{\beta}u^2,
\end{aligned}
\tag{9}
$$

in which $\tilde{\alpha}^{E1}$, $\tilde{\alpha}^{qm}$, and $\tilde{\beta}$ are the differential $E1$, $E2$-$M1$ polarizabilities, and hyperpolarizability on the clock transition, respectively. u is the lattice trap depth in units of E_{rec}, n_z is the axial vibrational quanta, and δ_L is lattice detuning from the effective magic wavelength, where the scalar and tensor shifts cancel. The vector shift is rejected by averaging the transitions with opposite nuclear spin states. Through clock sideband thermometry both axially and radially we find $n_z < 0.01$ and radial temperature $T_r < 200$ nK at $u_{op} = 15\ E_{\text{rec}}$, where thermal averaging of the effective trap depth can be neglected. By operating at lattice depths below 35 E_{rec}, the hyperpolarizability terms are also negligible.

To model the differential lattice light shift in our system, we introduce a dimensionless parameter δu, which characterizes the relative lattice trap depth difference between ensemble pair (i, j), where $j > i$

$$
\begin{aligned}
u_i &= u, \\
u_j &= u(1+\delta u).
\end{aligned}
\tag{10}
$$

For $\delta u \ll 1$, the differential light shift $(\nu_{LS,j} - \nu_{LS,i})$ can be approximated as

$$
\Delta\nu_{LS}(\delta_L,\delta u,u) = \delta u\left[\left(\frac{\partial\tilde{\alpha}^{E1}}{\partial\nu}\delta_L - \tilde{\alpha}^{qm}\right)\frac{1}{4}\sqrt{u} - \frac{\partial\tilde{\alpha}^{E1}}{\partial\nu}\delta_L u\right],
\tag{11}
$$

which scales linearly with δu.

By modulating the lattice detuning between $\delta_L + 100$ MHz and $\delta_L - 100$ MHz at the operational depth $u_{op} = 15\ E_{\text{rec}}$, we have

$$
\begin{aligned}
&\Delta\nu_{LS}(u_{op},\delta_L+100\text{ MHz}) - \Delta\nu_{LS}(u_{op},\delta_L-100\text{ MHz}) \\
&= \delta u\frac{\partial\tilde{\alpha}^{E1}}{\partial\nu}\left(\frac{1}{4}\sqrt{u_{op}} - u_{op}\right)\times 200\text{ MHz},
\end{aligned}
\tag{12}
$$

allowing extraction of δu, which are found to be below 5 % for all the ensemble pairs, and are symmetric around 0 (Supplementary Figure 4a), reflecting the Gaussian nature of the lattice beam profile and that the array is centered about the focus. Through mapping out the frequency shifts for 10 ensemble pairs when modulating the lattice intensity, we find the differential light shifts scale with the spatial separations between ensemble pairs but do not scale with δu, resulting in a residual spatial light shift gradient of $-8.0(1.1)\times10^{-20}/E_{\text{rec}}$/cm (Supplementary Figure 4b).

Although the origin of the spatial light shift gradient is not definitively known, we also observe a differential lattice vector Stark gradient of $-2.5(2)\times10^{-18}/E_{\text{rec}}$/cm by measuring the splitting between transitions with opposite spin states (Supplementary Figure 4c), which also does not scale with δu and instead scales with trap depth and spatial separation. For a single

ensemble, the vector Stark shift is proportional to $\xi_{\text{lat}}(\mathbf{e_k}\cdot\mathbf{e_B})$, where ξ_{lat} is the ellipticity of the lattice light, $\mathbf{e_k}$ is the lattice wave vector along $\mathbf{z}$ direction, and $\mathbf{e_B}$ is the magnetic field vector primarily along $\mathbf{x}$. We find the vector Stark gradient arises from the spatially varying coefficient, $\mathbf{e_k}\cdot\mathbf{e_B}$, verified through the change of vector Stark gradient by applying an additional magnetic field gradient $\partial B/\partial z$ of up to ± 10 mG/cm, which effectively changes $(\mathbf{e_k}\cdot\mathbf{e_{B,j}}-\mathbf{e_k}\cdot\mathbf{e_{B,i}})$. Based on this observation, we hypothesize that the residual spatial light shift gradient is due to a differential tensor Stark shift, in which the spatially varying magnetic field vectors are coupled to the (nearly) linear lattice polarization ($\mathbf{e}_{\text{lat}}$, along $\mathbf{x}$) through $|\mathbf{e}_{\text{lat}}\cdot\mathbf{e_B}|^{\mathbf{2}}$. The differential vector Stark shift results in a gradient of $-37(3)\times 10^{-18}$/cm at $u_{op}=15\ E_{\text{rec}}$ in the splitting between transitions with opposite spin states, equivalent to a fictitious magnetic field gradient of $\sim 600\ \mu$G/cm and is accounted for during the second-order Zeeman shift corrections.

To independently evaluate the light shift gradient, we need to account for the δu dependent shifts, which require the knowledge of δu and δ_L. The latter is done by first subtracting the residual spatial gradient from the measured differential light shifts. We then find that the remaining shifts are correlated with the extracted δu (Fig. 2d in the main text) as expected from Eq. 11. From this we find our operational lattice detuning δ_L to be 16.9(1.5) MHz. With the extracted δ_L and δu calibrated from each measurement day, we are able to monitor and account for the residual light shift gradient. A representative plot is shown in Fig. 2e in the main text.

Our operational lattice frequency is measured to be $368,554,780(30)$ MHz, limited by the accuracy of the wave-meter (HighFinesse, WS7). However, the lattice frequency is stabilized to a ultra-low-expansion cavity with typical drifts of less than -20 kHz per day, inferred from the narrow-linewidth ${}^1S_0 \leftrightarrow {}^3P_1$ MOT, providing sufficient long-term stability throughout the measurements. The lattice intensity is actively stabilized and controlled via feedback to the acoustic-optical modulator before the beam delivering optical fiber (NKT Photonics, LMA-PM-15). Both the incoming and retro-reflected lattice alignments are monitored on several cameras and photo-diodes, ensuring the daily-calibrated δu remained symmetric around 0 throughout the data taking campaign. Overall, the lattice light shift gradient in our system is evaluated to be $-11.8(1.2)\times 10^{-19}$/cm.

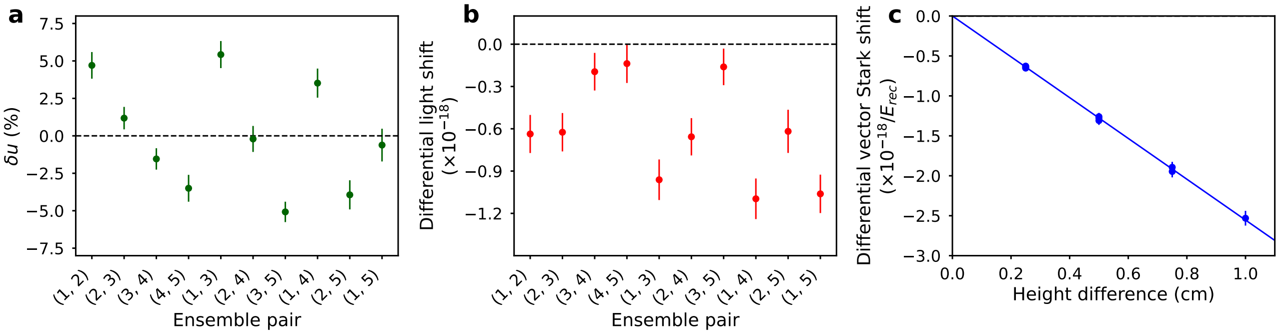

Supplementary Figure 4. Lattice light shift evaluation. (a) Extraction of relative trap depth difference δu for 10 ensemble pairs. The extracted δu are symmetric around 0, reflecting the Gaussian nature of the lattice beam profile and that the ensembles are centered about the beam focus. (b) Evaluation of differential lattice light shifts at $u_{op}=15\ E_{\text{rec}}$ for 10 ensemble pairs. The shifts scale with spatial separation, but do not scale with δu, resulting in a residual spatial lattice light shift gradient of $-8.0(1.1)\times 10^{-20}/E_{\text{rec}}$/cm. (c) Evaluation of the differential vector Stark shift, which also doesn't scale with δu. A linear fit (blue solid line) yields a gradient of $-2.5(2)\times 10^{-18}/E_{\text{rec}}$/cm.

E. Probe Stark shift

The probe AC Stark shift arises from the clock light itself, and is suppressed when probing with a shared clock light, with uncertainties primarily arising from inhomogeneity and misalignment of the clock beam with respect to the lattice. The clock light beam waist is ~ 1 mm, a factor of 10 greater than that of the lattice, ensuring homogeneity across the atomic ensembles both axially and radially. The clock light is carefully aligned to the lattice light, and is monitored using several cameras by picking-off the beam before and after the science chamber over a distance of ~ 2.5 m. The alignment is further verified by ensuring that the time periods of the clock transition Rabi oscillations of all 5 ensembles agree within 1% fitting uncertainty before and after each measurement run.

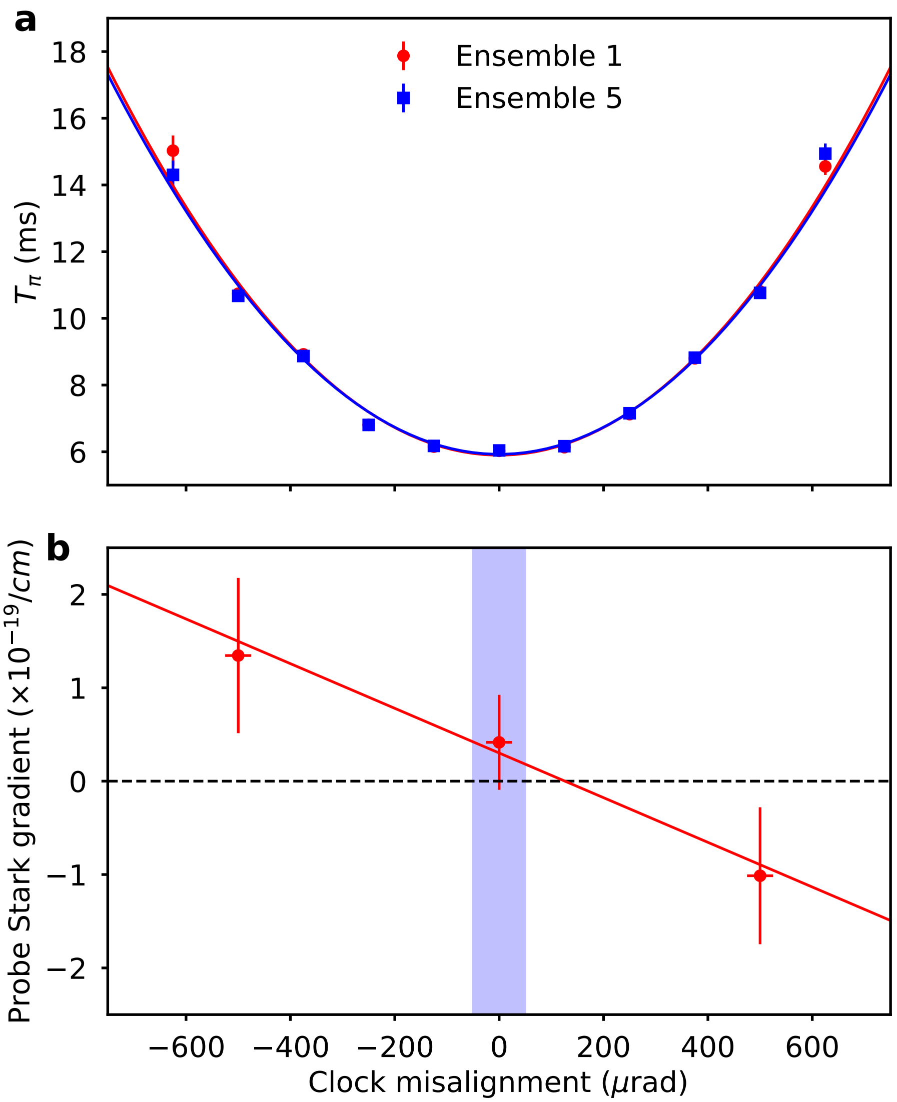

Supplementary Figure 5. Probe Stark shift evaluation. (a) Measured π pulse durations T_π for ensemble 1 (red) and 5 (blue), the pair with largest spatial separation (1 cm), as a function of the angle of clock beam misalignment. The solid lines are quadratic fit to the data. (b) Evaluated probe Stark shift at the operational intensity I_{op} at ± 500 μrad clock beam misalignment (estimated using camera). A linear fit to the data bounds the probe Stark shift gradient to be below 0.5×10^{-19}/cm (the blue area represents a ± 50 μrad range).

Evaluation of the probe Stark effect is performed by interleaving between the operational clock intensity of I_{op}, corresponding to a π pulse duration of 6 ms, and the case of $4I_{op}$. This interleaved measurement is repeated by further misaligning the clock beam by up to ±500 μrad (estimated via camera images), at which the π pulse durations increase by about a factor of 2 (Supplementary Figure 5). Through linear fitting, we find a frequency gradient of $2.5(1.0)\times10^{-19}$/cm across the span of ±500 μrad misalignments. Under normal operations, the misalignment is monitored and bounded within a ±50 μrad range which accounts for possible drifts during the experiments. This bounds the uncertainty from probe Stark shift to below 0.5×10^{-19}/cm.

F. DC Stark shift

Frequency shifts arising from electric fields can perturb the clock transition through DC Stark effect of the form kE^2, where $E = |\mathbf{E}|$ is the static electric field and $k \equiv -(\alpha_e - \alpha_g)/(2h)$ is the coefficient specific to the clock transition, with $\alpha_{g,e}$ being the static polarizabilities of the ground and excited clock states. To evaluate the DC Stark effect, a pair of quadrant electrodes are mounted along the lattice axis outside the top and bottom science chamber viewports, with a total separation of roughly 30 cm. We then probe the clock transition with opposite voltages applied to the electrode pair and interleave between $(+V, -V)$ and $(-V, +V)$ configurations. We observe differential shifts below the 1×10^{-19}/cm level when applying voltages of up to ±100 V to the electrodes, indicating that the background electric field gradient is small.

To quantify our uncertainty in the differential shifts due to the background electric field gradient, we follow the approach laid out in Ref. [11]. In the presence of a background field E_{bg}, which would most likely arise due to charge accumulation on the nearby top and bottom viewports, the DC Stark shift for a single atom ensemble is given by $k(E_{bg} + cV)^2$, where c is the atomic coefficient. We rewrite the above form as [11]

$$\nu_{dc}(V) = \nu_{bg} + aV + bV^2, \tag{13}$$

where ν_{bg} is the background stray field shift, and coefficients a and b are experimentally accessible parameters by modulating V. We note that aV represents the coupling between the background field and the applied field. When comparing ensemble i and ensemble j ($j > i$), the differential DC Stark shift ($\Delta\nu_{dc} = \nu_j - \nu_i$) becomes

$$\Delta\nu_{dc}(V) = \Delta\nu_{bg} + AV + BV^2, \tag{14}$$

where $A = a_j - a_i$, $B = b_j - b_i$, and $\Delta\nu_{bg} = \nu_{bg,j} - \nu_{bg,i}$ is the differential background shift due to charges on the viewports.

Due to the finite spatial extent (s) of the atom ensembles and inhomogeneity of the applied field, there is no V such that the applied field identically cancels $\Delta\nu_{bg}$. We consider the extremum value of $\Delta\nu_{dc}(V)$, denoted as $\Delta\nu^*$. We then have the difference between the differential background shift and the extremum shift, $\Delta\nu_{\text{diff}} \equiv \Delta\nu_{bg} - \Delta\nu^*$ (refer to Fig. 1 in Ref. [11] for details). From Eq. 14, we find $\Delta\nu_{\text{diff}} = A^2/(4B)$. We note that $\Delta\nu^*$ plays the role of a frequency correction for the field gradients, and can be written as $\Delta\nu^* = \eta\Delta\nu_{\text{diff}}$, due to the fact that $\Delta\nu^*$ and $\Delta\nu_{\text{diff}}$ scale similarly with the stray field. The differential background shift is then given by

$$\Delta\nu_{bg} = (1+\eta)\Delta\nu_{\text{diff}} = (1+\eta)A^2/(4B), \tag{15}$$

in which η is given by $\eta = \zeta^2 s^2/R^2$ to leading order of s, where $\zeta \equiv (q_1 + q_2)/(q_1 - q_2)$ quantifies the charge symmetry between the viewports, and R is the effect length.

By applying voltages of up to ±100 V to the electrodes, we find $A^2/(4B) = 0.6(3) \times 10^{-20}$ for the ensemble pair of 1 cm separation. In our system, we obtain $\eta \approx 0.02$ for $s =$ 500 μm, $R = 40$ mm, and assuming 25% more charge on one viewport than the other. This bounds the background DC Stark shift gradient to below 0.1×10^{-19}/cm.

G. Ellipse fitting

In the presence of QPN and numerical constraints which ensure an ellipse-specific solution, the least squares ellipse fitting approach is biased at phases close to 0 or π where the ellipse collapses into a straight line [12]. The differential phases across the ensemble array are dominated by the differential Zeeman shifts, such that through precise control of the magnetic field and gradient, we can operate in the regime where all the differential phases lie within the $(\pi/6, 5\pi/6)$ range. For typical experiments with a 10 s Ramsey dark time and 2000 atoms per ensemble, the ellipse fitting bias error is bound to below 2×10^{-19} (Supplementary Figure 6). During initial data processing, the bias error is corrected using Monte-Carlo simulations with the experiment parameters such as atom number and contrast as inputs. While the bias error does not scale with spatial separations, we estimate an upper bound limit of 0.5×10^{-19} due to uncertainty in the input parameters used for Monte-Carlo simulation during bias error corrections.

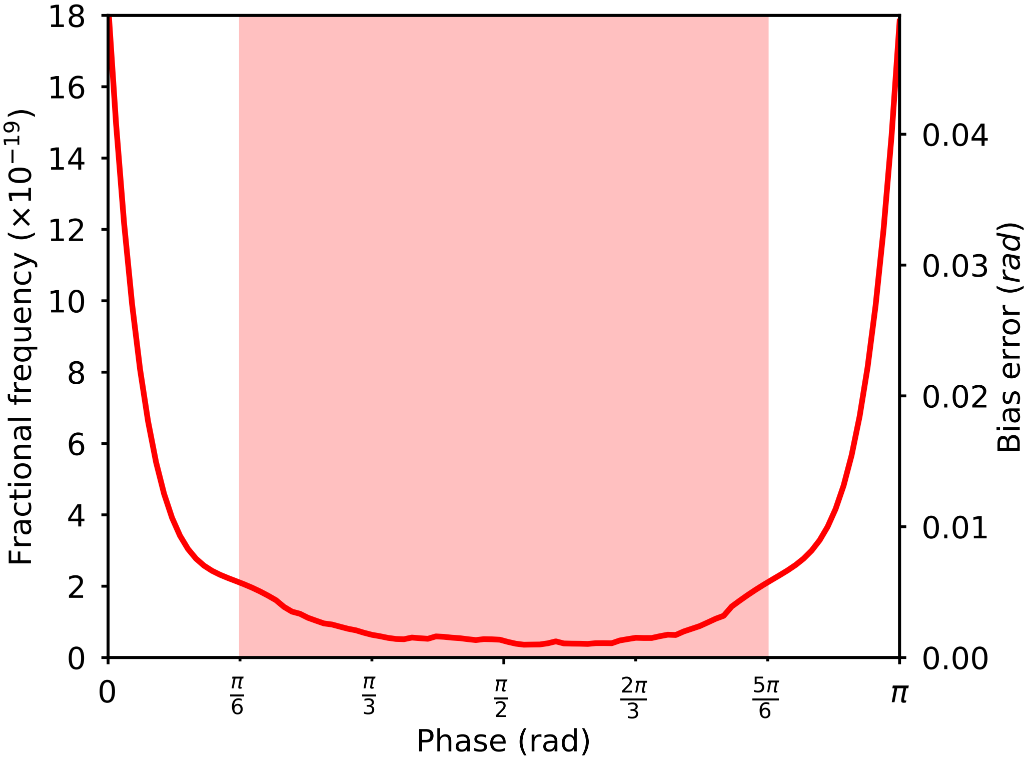

Supplementary Figure 6. Ellipse fitting bias error evaluation. Monte-Carlo simulations of bias error from ellipse fitting with input parameters of 2000 atoms per ensemble, 85% contrast and 10 s Ramsey dark time (red solid line). The red area represents our normal operating range of $(\pi/6, 5\pi/6)$, where fractional bias error is bound below 2×10^{-19} and is corrected for each ellipse fitting during data analysis with uncertainty below 0.5×10^{-19}.

H. Wannier-Stark ladder, imaging, and the expected gravitational redshift

The local gravitational acceleration in our laboratory was measured by the group of Prof. Tikoff from the Department of Geoscience at the University of Wisconsin-Madison using a LaCoste-Romberg gravimeter, and was then cross-validated with known values from several other survey points in Wisconsin. The local gravitational acceleration in our laboratory was measured to be $g = -9.803$ m/s^2, rounded to the 4th digit.

The lattice tilt (θ_{tilt}) with respect to gravity is independently measured using the splitting of the $\pm 1^{\text{st}}$ order Wannier-Stark sidebands [13] to the clock transition at 5 E_{rec} lattice depth (Supplementary Figure 7a), which is given by

$$\Delta\nu_{\text{WS},\pm 1} = mg\lambda_L \cos\theta_{\text{tilt}}, \tag{16}$$

where m is the mass of ^{87}Sr, and $\lambda_L = 813.4$ nm is the lattice wavelength. A representative plot of the measured Wannier-Stark sidebands is shown in Supplementary Figure 7b, and we find the weighted average splitting between $\pm 1^{\text{st}}$ order sidebands to be 1729(2) Hz, corresponding to a tilt with respect to gravity of 5.0(1)°.

The overall spatial extent of the ensemble array is 1.00(1) cm, calculated based on the frequency chirp profile of the moving optical lattice loading sequence, which is precisely controlled using a direct digital synthesizer (DDS, Moglabs XRF). This yields an effective height difference of $\Delta h = 0.99(1)$ cm, consistent with the extraction of the height difference from the camera images. The effective camera pixel size in our imaging system is calibrated to be 34(1) μm per pixel using the standard time-of-flight imaging.

Fluctuations of background magnetic fields modifying the narrow-line MOT operation could result in position drift in the atom ensemble. This is rejected by identifying the center-of-mass of each ensemble with a 2D Gaussian fit, followed by a selection of region-of-interest (ROI) with a 15 pixel by 15 pixel region centered on the fit. Each ensemble has a finite vertical spatial extent of roughly 500(10) μm, corresponding to an expected gradient of $-5.5(1) \times 10^{-20}$ across the ensemble due to the gravitational redshift, which is averaged out by spatial averaging over the ROI in order to extract the redshifts between the center-of-mass positions of each ensemble. The center-of-mass position uncertainty of each ensemble is limited by the DDS timing accuracy (100 ns), corresponding a position error of < 0.1 μm.

The expected gravitational redshift from theory of general relativity at a 1 cm height difference is then given by

$$\frac{g\Delta h}{c^2} = -10.9 \times 10^{-19}, \tag{17}$$

where c is the speed of light. This results in an expected redshift gradient of -10.9×10^{-19}/cm, with the uncertainty bounded $< 0.1 \times 10^{-19}$/cm due to uncertainties in g and Δh.

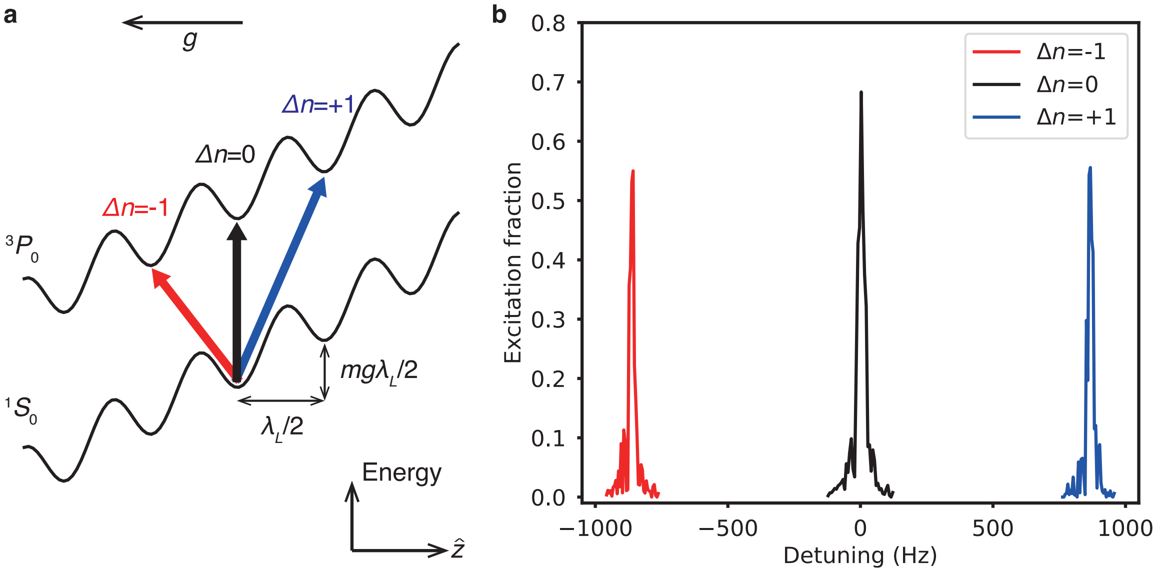

Supplementary Figure 7. Wannier-Stark ladder sidebands. (a) Illustration of the Wannier-Stark ladder in a 1D vertical optical lattice along the **z** direction. The Wannier state at lattice site n has energy $nmg\lambda_L/2$. The black arrow represents the carrier clock transition where n remains unchanged ($\Delta n = 0$). The blue (red) arrow represents the first-order sideband transition with $\Delta n = +1(-1)$. (b) Representative plot of measured Wannier-Stark sidebands at a shallow lattice depth (5 $E_{\rm rec}$) recorded with Fourier-limited Rabi spectroscopy with a π pulse duration of 40 ms. The black line corresponds to the carrier ($\Delta n = 0$) transition, and the blue (red) line corresponds to off-site transitions, $\Delta n = +1(-1)$. The splitting between $\Delta n = \pm 1$ transitions is used to extract the lattice tilt.

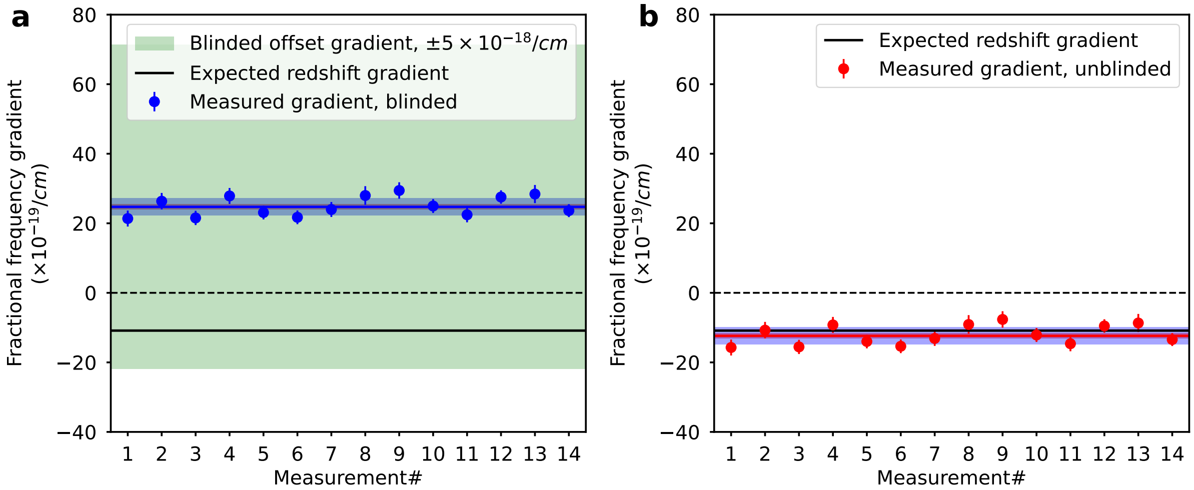

Supplementary Figure 8. Measurements before and after removal of the blinded offset gradient. (a) Measured fractional frequency gradients (blue scatter points) with blinded offset. The blue area represents $\pm 1\sigma$ total uncertainty. The black solid line is the expected gravitational redshift gradient of -10.9×10^{-19}/cm. The green area represents the range of possible outcomes upon removal of the blinded offset gradient, corresponding to a $\pm 5 \times 10^{-18}$/cm range. All corrections and uncertainties were finalized at this stage, without knowledge of the blinded offset. (b) Measured fractional frequency gradients following removal of the blinded offset. Red scatter points are the same data set as in (a) with the blinded offset gradient ($+3.7 \times 10^{-18}$/cm) removed. This represents the same data shown in Fig. 3a in the main text, but here the scale of the y-axis is kept the same as in panel (a) for the sake of comparison.

	Measured gravitational redshift ($\times 10^{-19}$)	Measured height difference (cm)	Expected height difference (cm)
Pair (1,2)	-2.71(1.98)	0.25(18)	0.25(1)
Pair (2,3)	-4.68(1.99)	0.43(18)	0.25(1)
Pair (3,4)	-2.04(1.96)	0.19(18)	0.25(1)
Pair (4,5)	-2.52(2.02)	0.23(19)	0.25(1)
Pair (1,3)	-7.23(2.18)	0.66(20)	0.50(1)
Pair (2,4)	-5.78(2.19)	0.53(20)	0.50(1)
Pair (3,5)	-6.22(2.19)	0.57(20)	0.50(1)
Pair (1,4)	-7.91(2.52)	0.73(23)	0.75(1)
Pair (2,5)	-8.90(2.50)	0.82(23)	0.75(1)
Pair (1,5)	-11.96(2.93)	1.10(27)	1.00(1)

Supplementary Table 1. Clock height difference measurement. The measured gravitational redshift of each clock comparison is used to extract the height difference given the independently measured local gravitational acceleration, $g = -9.803$ m/s^2.

References

[1] Zheng, X. et al. Differential clock comparisons with a multiplexed optical lattice clock. *Nature* **602**, 425–430 (2022).

[2] Boyd, M. M. et al. Nuclear spin effects in optical lattice clocks. *Phys. Rev. A* **76**, 022510 (2007).

[3] Martin, M. J. et al. A quantum many-body spin system in an optical lattice clock. *Science* **341**, 632–636 (2013).

[4] Zhang, X. et al. Spectroscopic observation of SU(N)-symmetric interactions in Sr orbital magnetism. *Science* **345**, 1467–1473 (2014).

[5] Aeppli, A. et al. Hamiltonian engineering of spin-orbit–coupled fermions in a Wannier-Stark optical lattice clock. *Sci. Adev.* **8**, adc9242 (2022).

[6] Boyd, M. M. *Ph. D. thesis* (2013).

[7] Beloy, K. et al. Atomic clock with 1×10^{-18} room-temperature blackbody stark uncertainty. *Phys. Rev. Lett.* **113**, 260801 (2014).

[8] Ushijima, I., Takamoto, M., Das, M., Ohkubo, T., & Katori, H. Cryogenic optical lattice clocks. *Nat. Photon.* **9**, 185–189 (2015).

[9] Haslinger, P., Jaffe, M., Xu, V., Schwartz, O., Sonnleitner, M., Ritsch-Marte, M., Ritsch, H., & Müller, H. Cryogenic optical lattice clocks. *Nat. Photon.* **9**, 185–189 (2015).

[10] Ushijima, I., Takamoto, M. & Katori, H. Operational magic intensity for Sr optical lattice clocks. *Phys. Rev. Lett.* **121**, 263202 (2018).

[11] Beloy, K. et al. Faraday-shielded dc stark-shift-free optical lattice clock. *Phys. Rev. Lett.* **120**, 183201 (2018).

[12] Halir, R. & Flusser, J. Numerically stable direct least squares fitting of ellipses. *Proc. of Sixth Intl Conf. Computer Graphics and Visualization* **1** (1998).

[13] Lemonde, P. & Wolf, P. Optical lattice clock with atoms confined in a shallow trap. *Phys. Rev. A* **72**, 033409 (2005).
